# Dehydrogenation Performances of Different Al Source Composite Systems of 2LiBH_4_ + M (M = Al, LiAlH_4_, Li_3_AlH_6_)

**DOI:** 10.3389/fchem.2020.00227

**Published:** 2020-04-15

**Authors:** Yun Li, Shaolong Wu, Dongdong Zhu, Jun He, Xuezhang Xiao, Lixin Chen

**Affiliations:** ^1^School of Mechanical and Electrical Engineering, Quzhou College of Technology, Quzhou, China; ^2^Key Laboratory of Advanced Materials and Applications for Batteries of Zhejiang Province, Zhejiang University, Hangzhou, China; ^3^School of Mechanical Engineering, Quzhou University, Quzhou, China; ^4^Quzhou Special Equipment Inspection Center, Quzhou, China

**Keywords:** hydrogen storage materials, LiBH_4_, composite system, 2LiBH_4_-Li_3_AlH_6_, dehydrogenation performance

## Abstract

Hydrogen has become a promising energy source due to its efficient and renewable properties. Although promising, hydrogen energy has not been in widespread use due to the lack of high-performance materials for hydrogen storage. Previous studies have shown that the addition of Al-based compounds to LiBH_4_ can create composites that have good properties for hydrogen storage. In this work, the dehydrogenation performances of different composite systems of 2LiBH_4_+ M (M = Al, LiAlH_4_, Li_3_AlH_6_) were investigated. The results show that, under a ball to powder ratio of 25:1 and a rotation speed of 300 rpm, the optimum ball milling time is 50 h for synthesizing Li_3_AlH_6_ from LiH and LiAlH_4_. The three studied systems destabilized LiBH_4_ at relatively low temperatures, and the 2LiBH_4_-Li_3_AlH_6_ composite demonstrated excellent behavior. Based on the differential scanning calorimetry results, pure LiBH_4_ released hydrogen at 469°C. The dehydrogenation temperature of LiBH_4_ is 416°C for 2LiBH_4_-Li_3_AlH_6_ versus 435°C for 2LiBH_4_-LiAlH_4_ and 445°C for 2LiBH_4_-Al. The 2LiBH_4_-Li_3_AlH_6_, 2LiBH_4_-LiAlH_4_, and 2LiBH_4_-Al samples released 9.1, 8, and 5.7 wt.% of H_2_, respectively. Additionally, the 2LiBH_4_-Li_3_AlH_6_ composite released the 9.1 wt.% H_2_ within 150 min. An increase in the kinetics was achieved. From the results, it was concluded that 2LiBH_4_-Li_3_AlH_6_ exhibits the best dehydrogenation performance. Therefore, the 2LiBH_4_-Li_3_AlH_6_ composite is considered a promising hydrogen storage material.

## Introduction

Hydrogen energy has become an ideal new energy resource due to its clean, efficient, and renewable properties (Schlapbach and Zuttel, 2001). Although the use of hydrogen energy is promising, widespread use has been hindered by issues in the advancement of high-performance materials for hydrogen storage (Liu et al., 2010). The complex hydride (Ley et al., 2014) LiBH_4_, which has a hydrogen capacity storage of 18.5 wt.%, has drawn extensive attention. LiBH_4_ decomposes into an intermediate compound Li_2_B_12_H_12_, which generates B and releases H_2_ as described in Equation (1) (Orimo et al., 2006).

(1) $$\begin{array}{lll} \text{LiB}{\text{H}}_{4} & \to & 1/12\text{L}{\text{i}}_{2}{\text{B}}_{12}{\text{H}}_{12}+5/6\text{LiH}+13/12{\text{H}}_{2}\to \text{LiH}\\ \;+ & \text{B}+3/2{\text{H}}_{2} \end{array}$$

However, many factors impede the commercial application of pure LiBH_4_, such as high dehydrogenation temperature, slow dehydrogenation rate, and poor cycle reversibility (Lodziana and Vegge, 2004). To solve these issues, researchers have concentrated on modifying LiBH_4_ (Vajo and Olson, 2007) through anion/cation substitution (Fang et al., 2011; Lombardo et al., 2019), catalytic modification (Kou et al., 2012; Huang et al., 2016; Zhai et al., 2016), the combined effect of composites (Vajo et al., 2010; Kou et al., 2012), and the application of the confinement effect of nano-materials (Vajo, 2011; Zhang et al., 2017).

Al and Al-based composites with LiBH_4_ are of interest. Founded on first-principles calculation, Siegel et al. (2007) predicted that LiBH_4_ would react with Al to generate AlB_2_, LiH, and H_2_ at 280°C under a hydrogen pressure of 1 bar. For the LiAlH_4_-LiBH_4_ system, Mao et al. (2009) found that the addition of TiF_3_ decreased the onset temperatures of H_2_ release by 64 and 150°C compared with the undoped system. The decomposition enthalpy values of LiBH_4_ also reduced to 60.4 kJ/mol. He et al. (2019) studied the dehydrogenation performance of LiBH_4_/LiAlH_4_ composite, found that 8.7 wt.% of hydrogen was released at 500°C, and defined a “Li-Al-B-H” compound. Soru et al. (2014) focused on the phase structural transformation of the LiAlH_4_ + LiBH_4_ system, which can produce 6.8 wt.% of hydrogen. According to a study by Carrillo-Bucio et al. (2017), a surface-oxidized 2LiBH_4_ + Al composite did not release hydrogen until heated to 400°C under a 3 bar initial backpressure. With the catalytic effect of TiF_3_, the mixture obtained 9.3 wt.% of hydrogen release as compared to the 5.8 wt.% of the undoped mixture. In research by Zhang et al. (2018), a combined mixture of MgH_2_, LiBH_4_, and LiAlH_4_ showed superior performance, starting to release hydrogen at 280°C and maintaining reversibility. In our previous studies (Li et al., 2012), the dehydrogenation temperatures of 2LiBH_4_ + Li_3_AlH_6_ doped with titanium were decreased by 80 and 50°C, respectively, vs. the undoped system. From the previous studies, it can be summarized that Al can enhance the hydrogen storage performance of LiBH_4_ to some extent. However, the dehydrogenation behaviors of different Al source composite systems have not been systematically studied. In this work, three Al-based LiBH_4_ composite systems, 2LiBH_4_-Al, 2LiBH_4_-LiAlH_4_, and 2LiBH_4_-Li_3_AlH_6_ were prepared, and the hydrogen storage performance was investigated.

## Materials and Methods

Table 1 shows the raw materials used in this study. All powders were carefully stored in a glove box (MIKROUNA), in which noble gas was loaded and the oxygen content and water vapor content were kept below 1 ppm. A planetary mill (QM-3SP2) was utilized to prepare the composites.

**Table 1:** Raw materials used in the study.

**Materials**	**LiBH_**4**_**	**Al**	**LiAlH_**4**_**	**LiH**
Purity (%)	≥95	≥99	≥95	≥98
State	Powder	Powder	Powder	Powder
Supplier	Acros	Sinopharm	Sigma-Aldrich	Sigma-Aldrich

The dehydrogenation properties of the materials were tested with a Sievert-type device. The changes in temperature and pressure over time were recorded. The ideal gas state equation was utilized to calculate the dehydrogenation capacity. Thermal analysis was performed using differential scanning calorimetry (DSC, Netzsch 449C Jupiter) combined with thermogravimetric analysis (TG, QMS 403C). To protect the samples from oxidation, high purity argon gas was added at a flow rate of 50 mL/min. An empty aluminum crucible was used as a reference during analysis. The phase compositions of the samples were determined by X-ray powder diffraction (X'Pert-PRO, PANalytical); its scanning range (2θ) was 15°-80°. A specific sealed device was used to protect samples from being oxidized or damped during analysis. Fourier transform infrared spectroscopy (FTIR, Tensor 27) was employed to detect some amorphous substances with a scanning rate of 30 cm^−1^/min and a resolution ratio of 0.5 cm^−1^.

## Results and Discussion

### Preparation of Li_3_AlH_6_ Powder

**Figure 1:**
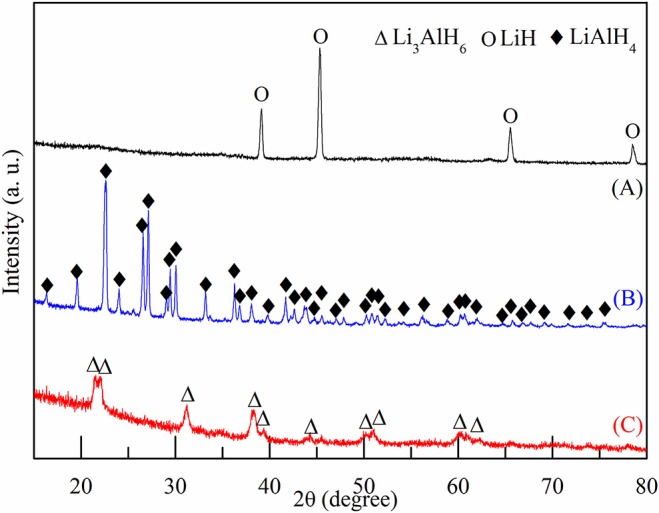
XRD patterns of **(A)** pure LiH, **(B)** pure LiAlH_4_, and **(C)** mixed powder of LiH and LiAlH_4_ after ball milling for 20 h.

In this work, Li_3_AlH_6_ powder was synthesized *in-situ* by ball milling LiH and LiAlH_4_. Approximately 15 g mixed powder of LiH and LiAlH_4_ was milled each time with a molar ratio of 2:1, a ball to powder ratio of 25:1, and a milling speed of 300 rpm. To suppress temperature rise in the ball milling, every 30 min was set as an operation cycle, which contained a stop time of 6 min, periodically. After ball milling for 20 h, XRD patterns were obtained and examined to characterize the mixed powder. Figure 1 shows the XRD results of pure LiH powder, LiAlH_4_ powder, and the LiH and LiAlH_4_ powder after ball milling for 20 h. No impurity phases were observed in any of the samples, which indicates that the samples were highly pure and did not oxidize. As shown in Figure 1C, a few Li_3_AlH_6_ diffraction peaks are present, while the rest are of LiAlH_4_ and LiH. This shows that the synthesis reaction was not completed. Due to long milling time, LiH peaks were much lower than before. The LiH content was lower, and an amorphous structure was formed.

**Figure 2:**
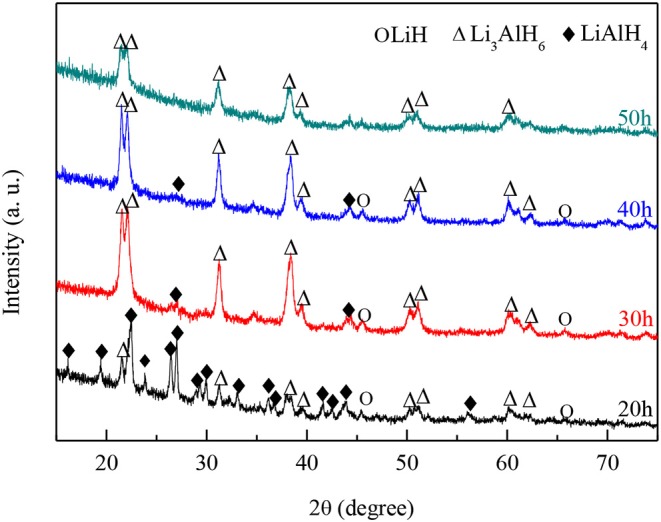
XRD patterns of mixed powder of LiH and LiAlH_4_ after ball-milling for different numbers of hours.

Further ball milling was carried out since the synthesis reaction to Li_3_AlH_6_ was not completed. The milling samples were taken for XRD analysis after every 10 h. Figure 2 shows the XRD results of the milled powders after 20, 30, 40, and 50 h. Strong double peaks at 22° and 23° are detected, which are the characteristic peaks of Li_3_AlH_6_. After ball milling for 30 h, most of the diffraction peaks are Li_3_AlH_6_; however, some LiAlH_4_ and LiH peaks can still be found at 27°, 43°, and 46°. As the ball milling time increased, the LiH and LiAlH_4_ content decreased, which shows that the synthesis reaction proceeds as the milling time increases. Nevertheless, the diffraction intensity of Li_3_AlH_6_ decreased, which shows that the longer milling time, the more likely the production of an amorphous phase becomes.

**Figure 3:**
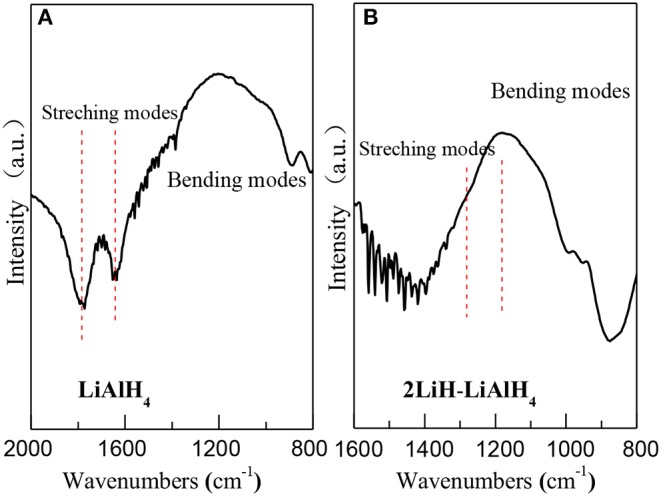
FT-IR spectrums of **(A)** pure LiAlH_4_ and **(B)** 2LiH-LiAlH_4_ mixed powder after 50-h ball milling.

FTIR analysis was used to investigate the synthesized powder. In pure LiAlH_4_ (Figure 3A), the bending modes around 1,780 and 1,610 cm^−1^ correspond to the A-H stretching band, as was previously shown by Chen et al. (2001). After 50 h ball milling, this A-H stretching band disappeared in the 2LilH-LiAlH_4_ mixtures (Figure 3B). A new A-H stretching band was generated around 1,380 cm^−1^, which belongs to Li_3_AlH_6_. This indicates the completion of the synthesis reaction.

### Characterization of the Composite Samples

**Figure 4:**
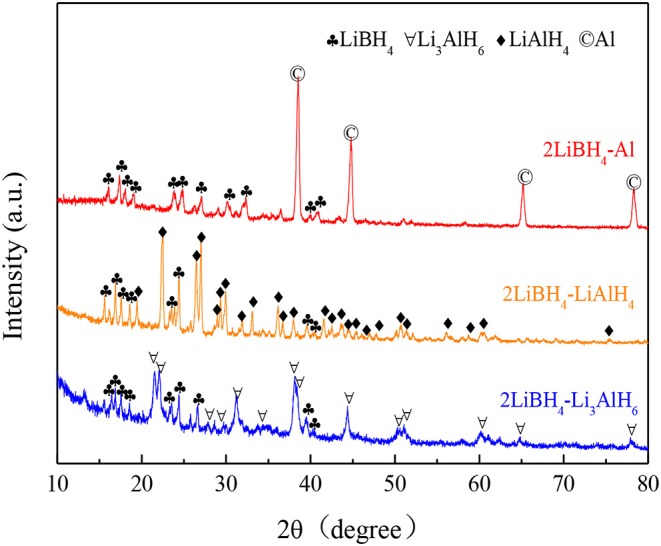
XRD patterns of 2LiBH_4_-Al, 2LiBH_4_-LiAlH_4_, and 2LiBH_4_-Li_3_AlH_6_ mixtures after ball milling for 1 h.

The 2LiBH_4_ + M (M = Al, LiAlH_4_, Li_3_AlH_6_) composite systems were prepared by ball milling, respectively. The ball-milling time was 1 h, with a mole ratio of 2:1. The as-milled mixtures are presented in Figure 4. In the 2LiBH_4_-Al composite, the narrow and sharp diffraction peaks are of Al, while the other diffraction peaks are of LiBH_4_. The intensity of the LiAlH_4_ peaks in the 2LiBH_4_-LiAlH_4_ composite are also strong, but both LiBH_4_ and Li_3_AlH_6_ have attenuated diffraction peaks in 2LiBH_4_-Li_3_AlH_6_. The absence of other peaks indicated that there were no side reactions that could generate impurities or by-products during ball milling.

### Dehydrogenation Performances

**Figure 5:**
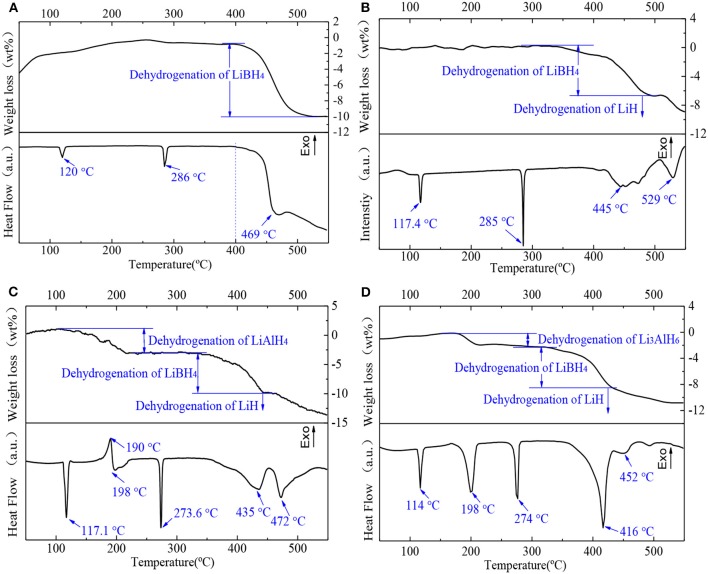
DSC/TG curves of **(A)** pure LiBH_4_, **(B)** 2LiBH_4_-Al, **(C)** 2LiBH_4_-LiAlH_4_, and **(D)** 2LiBH_4_-Li_3_AlH_6_ samples after ball milling for 1 h.

The dehydrogenation performances of the samples were investigated. The four samples were heated to 550°C at a heating rate of 5°C/min in an argon atmosphere. The DSC/TG results are shown in Figure 5. In the pure LiBH_4_ sample (Figure 5A), the endothermic peak at 120°C denotes that LiBH_4_ transformed from an orthorhombic to a hexagonal crystal system. The endothermic peak at 286°C is attributed to the melting of LiBH_4_. According to the TG curve, LiBH_4_ began to release hydrogen and lose weight at 400°C. There is a dehydrogenation peak at around 469°C, and the rate of dehydrogenation slowed down after that. Both the transformation and the melting peak of LiBH_4_ for each composite sample are shown in Figures 5B–D. All samples have lower transformation and melting temperatures than pure LiBH_4_ (transformation temperature: *T*_d_ < *T*_c_ < *T*_b_ < *T*_a_; melting temperature: *T*_c_ < *T*_d_ < *T*_b_ < *T*_a_). In the as-milled 2LiBH_4_-Al sample (Figure 5B), the wide endothermic range around 445°C represents the decomposition of LiBH_4_. The last peak at 529°C is the decomposition of LiH. As shown in Figure 5C, peaks at 190 and 198°C indicated the dehydrogenation of LiAlH_4_, which were verified in our previous study (Li et al., 2012). LiAlH_4_ decomposed into Li_3_AlH_6_ at 190°C, and Li_3_AlH_6_ started to generate LiH, Al, and release H_2_ at 198°C. LiBH_4_ and LiH started to decompose at 435 and 472°C, respectively. Figure 5D shows that the decomposition peak of Li_3_AlH_6_ is at 198°C, which is consistent with the 2LiBH_4_-LiAlH_4_ sample. The decomposition peak of LiBH_4_ (416°C) is lower than that of the 2LiBH_4_-LiAlH_4_ (435°C) or 2LiBH_4_-Al samples (445°C). In addition, the decomposition peak of LiH, which is around 452°C, is also lower than that of the other composite systems. These results showed that the LiBH_4_ became more unstable due to the addition of Li_3_AlH_6_ and that hydrogen was released at a lower temperature. The main reason for this is that the Al present in Li_3_AlH_6_ was active enough to stimulate the dehydrogenation reaction.

**Figure 6:**
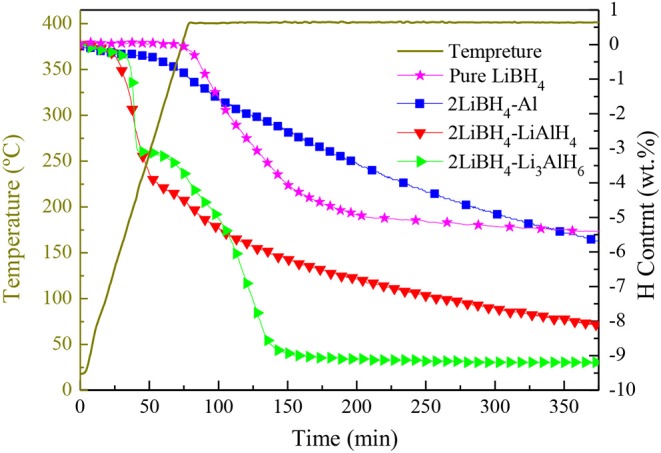
TPD curves of pure LiBH_4_, 2LiBH_4_-Al, 2LiBH_4_-LiAlH_4_, and 2LiBH_4_-Li_3_AlH_6_ samples.

The temperature-programmed desorption (TPD) method was implemented to study the dehydrogenation performance of pure LiBH_4_, 2LiBH_4_-Al, 2LiBH_4_-LiAlH_4_, and 2LiBH_4_-Li_3_AlH_6_; the results are shown in Figure 6. The samples were heated to 400°C and held for 5 h. The pure LiBH_4_ began to release H_2_ at around 400°C. Its desorption rate slowed down when 5 wt.% of H_2_ had been released. The 2LiBH_4_-Li_3_AlH_6_ sample released 9.1 wt.% H_2_ within 150 min, which is the fastest reaction kinetics in this work. Its decomposition process includes two main steps, with the release of 3 and 6.1 wt.% H_2_ as the first and second steps, respectively. In the 2LiBH_4_-LiAlH_4_ sample, LiAlH_4_ began to decompose at ~130°C and released 3.9 wt.% H_2_. Subsequently, its desorption rate was retarded. This composite released a total of 8 wt.% H_2_. The 2LiBH_4_-Al composite had a slower desorption rate. Only 5.7 wt.% H_2_ was released after 6 h, which is much lower than the 8.6 wt.% predicted by theoretical capacity. Previous research (Friedrichs et al., 2009) has reported that Al is an effective catalyst to activate LiBH_4_, and this phenomenon can also be observed in this study. However, the Al element generated from 2LiBH_4_-Li_3_AlH_6_ is much more effective than for the other samples. Since aluminum can be easily oxidized, an oxide film can easily form on the surface of the Al powder, which can slow down the dehydrogenation kinetics. In contrast, the Al in 2LiBH_4_-Li_3_AlH_6_, decomposed from Li_3_AlH_6_ exhibits high purity and is non-oxidized, resulting in the superior reaction kinetics of LiBH_4_.

### Characterization of the Dehydrogenation Materials

**Figure 7:**
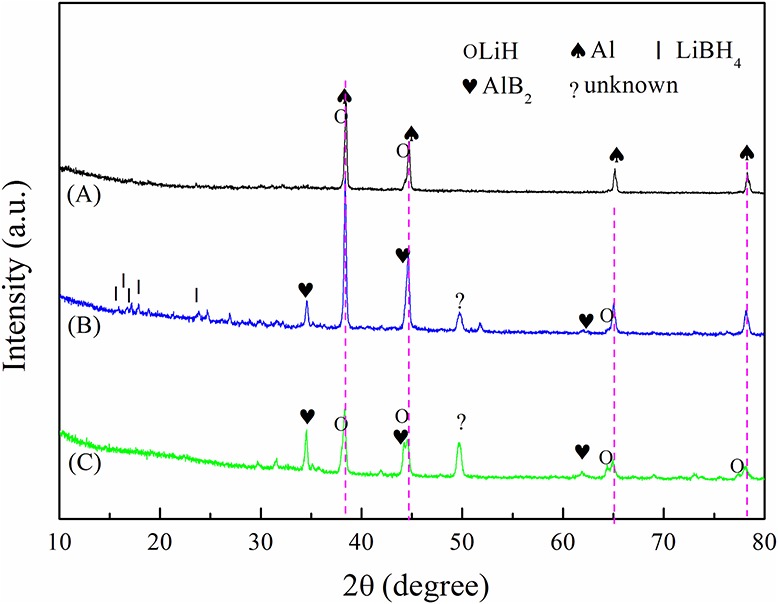
XRD results of dehydrogenated samples at 400°C: **(A)** 2LiBH_4_-Al, **(B)** 2LiBH_4_-LiAlH_4_, and **(C)** 2LiBH_4_-Li_3_AlH_6_.

The XRD patterns of the dehydrogenated samples are shown in Figure 7. In Figure 7A, prominent diffraction peaks of LiH and Al are present. However, the LiBH_4_ phase was not observed, as its diffraction intensity is relatively weaker. In Figure 7B, residual peaks of LiBH_4_ are observed, which demonstrates incomplete dehydrogenation. In Figure 7C, besides LiH and Al, the dehydrogenation products contain AlB_2_, which was reported as the reversible phase (Friedrichs et al., 2009). AlB_2_ can accelerate the formation of LiBH_4_ in the reverse reaction. The AlB_2_ content was higher in 2LiBH_4_-Li_3_AlH_6_ than in the other samples, which signifies higher reversibility of 2LiBH_4_-Li_3_AlH_6_. There is an unexpected peak near 50° both in the 2LiBH_4_-LiAlH_4_ and 2LiBH_4_-Li_3_AlH_6_ samples, which was not identified but was also detected in previous reports (Yang et al., 2007).

## Conclusions

In this work, the dehydrogenation performance of three different Al source composite systems, 2LiBH_4_-Al, 2LiBH_4_-LiAlH_4_, and 2LiBH_4_-Li_3_AlH_6_, was analyzed. Elemental Al was the raw material and was used without further purification in the 2LiBH_4_-Al composite, in contrast to the other two samples, where it was decomposed from LiAlH_4_ or Li_3_AlH_6_. Li_3_AlH_6_ powder was prepared from LiH and LiAlH_4_. It can be concluded that the optimum synthetic conditions for ball milling are 50 h with a 25:1 ball to powder ratio at 300 rpm. The results demonstrate that Al, LiAlH_4_, and Li_3_AlH_6_ had a stimulative effect on LiBH_4_, allowing dehydrogenation at a relatively lower temperature. Additionally, 2LiBH_4_-Li_3_AlH_6_ was shown to have the best performance, with the endothermic peak of LiBH_4_ at 416°C, 53°C lower than that of the pure LiBH_4_ sample (469°C). The TPD results also verified the superior results of 2LiBH_4_-Li_3_AlH_6_, which showed the best kinetics performance among the composite samples. 2LiBH_4_-Li_3_AlH_6_ released 9.1 wt.% H_2_ in only 150 min, which is over 95% of its theoretical hydrogen storage capacity. Its dehydrogenation product, AlB_2_, was reported as a reversible phase by researchers (Friedrichs et al., 2009), which could promote the reverse reaction of producing LiBH_4_. Further studies are needed to research the reversibility of the 2LiBH_4_-Li_3_AlH_6_ composite.

## Data Availability Statement

All datasets generated for this study are included in the article/supplementary material.

## Author Contributions

LC and YL: conception and design of study. YL and JH: acquisition of data. YL and XX: analysis and/or interpretation of data. YL: drafting the manuscript. DZ and SW: revising the manuscript critically for important intellectual content. YL, SW, DZ, JH, XX, and LC: approval of the version of the manuscript to be published.

### Conflict of Interest

The authors declare that the research was conducted in the absence of any commercial or financial relationships that could be construed as a potential conflict of interest.
